# Introducing Neurosurgery 2.0

**DOI:** 10.4103/2152-7806.63900

**Published:** 2010-05-31

**Authors:** Pieter L. Kubben

**Affiliations:** Neurosurgery 2.0 Editor, Department of Neurosurgery, Maastricht University Medical Center, The Netherlands

Before introducing “Neurosurgery 2.0” I will briefly introduce myself. My name is Pieter Kubben, and I work as a senior resident in neurosurgery at the Maastricht University Medical Center, The Netherlands. Besides my clinical activities I participate in two areas related to information technology. The first area is my PhD thesis focusing on intraoperative MRI. The results of the first study have been submitted for publication, and I expect the results of the second study to be submitted later this year. The second area is computer programming for the web and mobile devices, focusing on clinical decision support systems. After two international awards for my past efforts, I am currently preparing more interactive applications that share the Web 2.0 philosophy.

## WEB 2.0

To explain in detail what every neurosurgeon should know about the Web 2.0 would take a separate editorial: this will be the topic for the next issue. To give you a head start, Web 2.0 is an online paradigm shift from “publishing” towards “participation”: users add value to the web while using it. Access to web services is not limited to desktop computers, but involves a broad range of mobile devices. Think of Wikipedia, weblogs, Twitter, podcasts and YouTube as some general examples. If you are not familiar with these, simply ignore them for now. In any case, comparable web services can be developed for medical purposes as well.

## MEDICINE 2.0

The term “Medicine 2.0” can be found in some articles, and refers to the implementation of Web 2.0 techniques in healthcare. It facilitates medical knowledge sharing to support healthcare professionals in doing their work, or patients in getting informed. Related terms are “Health 2.0” and “e-Health”, but they do not have a clear distinction or precise definition. All can include telemedicine, or even doctor-patient communication.

Why should we welcome Medicine 2.0? Because of many reasons, but for now I will focus on one: the new methods it offers for sharing knowledge that can help physicians to learn about the latest developments in their specialty. To mention a few:

1. Web-based video and podcasting can use multimedia to produce more vivid and perhaps more time-effective methods of information delivery. They can summarize paper findings or offer additional details or insights.
2. Weblogs and forums can connect physicians to discuss difficult cases or share tips on technical procedures more informally.
3. Wikis and other collaborative communities can serve as knowledge resources that are critically reviewed and kept up to date by peer group members.

With more than 700,000 articles currently added to Medline every year, it is practically impossible to read all relevant information. Even finding that information is a challenge. For that reason, harnessing collective intelligence cannot be seen as “optional” anymore. Open-access resources can offer bridges to connect many content experts and knowledge resources, without financial restraints limiting participation. This is the goal of *Surgical Neurology International*, and “Neurosurgery 2.0” is one of the approaches that will be used.

## NEUROSURGERY 2.0

Neurosurgery 2.0 will be an online platform to provide Web 2.0 services for neuroscience in general, and neurosurgery in particular. The main goal of “Neurosurgery 2.0” will be to support bridging the “know-do gap” by offering web-based clinical decision support systems that run on a wide variety of computers, including mobile devices. We will start with offering a decision support system that is currently being developed. Then we will proceed with new decision support systems by gradually integrating your requests and needs in a Web 2.0 approach.

## WHERE TO START?

Here comes the key question: where to start? I will not misuse this editorial to share my enthusiasm for web development techniques like CSS, JQuery or server-side scripting. Those who are interested in technical details, please visit my weblog (DigitalNeurosurgeon.com). Instead I intend to use this editorial to invite you to share your opinion. *Surgical Neurology International* is an open-access journal for all neurosurgeons, neuroscientists and residents worldwide. So I consider “think global, act local” as the best way *how* to start. Where to start, depends on your needs and the data available. Recently, I released two mobile decision support systems: one for the surgical treatment of subaxial cervical spine injury, and one based on the “WHO Safe Surgery” checklist. Currently, I am finishing a web-based decision support system on peri-operative anticoagulation based on a Dutch review. The decision support system will be translated into English and placed on the website for you to use and evaluate. For the future I am interested in learning about clinical reviews or guidelines that cover topics of your interest. These should be relevant for a large international group of neurosurgeons, and subsequent decision support systems should help to improve patient outcome. In personal communication, “brain trauma” frequently came up in discussions, and the Brain Trauma Foundation did an excellent job in providing access to relevant guidelines. Personally, I consider these as a good place to start, but I would like to hear your opinion on this.

Any suggestions are welcome! Where would you start?

